# Technological and nutritional properties of meat from female wild boars (*Sus scrofa scrofa* L.) of different carcass weights

**DOI:** 10.5194/aab-62-597-2019

**Published:** 2019-12-17

**Authors:** Anna Kasprzyk, Joanna Stadnik, Dariusz Stasiak

**Affiliations:** 1 Department of Pig Breeding and Biotechnology, Institute of Animal Breeding and Biodiversity Conservation, University of Life Sciences in Lublin, Akademicka 13, 20-950 Lublin, Poland; 2 Department of Animal Raw Materials Technology, Faculty of Food Science and Biotechnology, University of Life Sciences in Lublin, Skromna 8, 20-704 Lublin, Poland

## Abstract

The aim of this work was to assess the technological and nutritional quality of meat from female wild boars. The muscle samples – Musculus longissimus thoracis et lumborum (LTL) and Musculus semimembranosus (SM) – were taken from a total of 40 female wild boar after a hunt. Carcasses were allocated to five groups according to weight (group I – 30±5 kg; group II – 45±4.9 kg; group III – 60±4.7 kg; group IV – 75±5.2 kg; group V – 90±5 kg). Studies that have been carried out have shown that technological and nutritional properties of meat from wild boars depend on the mass of carcasses and the type of muscle. The pH of analyzed wild boar meat proved that there was normal glycolysis and glycogenolysis progress in all groups. The water holding capacity (WHC) of SM muscles from the lowest-weight carcasses was significantly (P≤0.01) lower as compared to the heavier carcasses. There were significant differences (P≤0.01) in the shear force of the LTL muscle between groups I, IV and V. The muscles cut from carcasses of a higher mass represent higher values of this parameter. The higher-mass carcasses were characterized by a darker color, which resulted from the higher concentration of myoglobin. The protein concentration increased with carcass weight. A similar effect of carcass weight on the content of intramuscular fat (IMF) was found. Due to the low calorie content, the meat of young wild boar may be an interesting and attractive component of the diet.

## 1 Introduction

Healthy diets characterized by low calories and low cholesterol levels with the appropriate taste, juiciness and tenderness of meat products are a very important part of today's life style preferences of modern consumers (Hoffman and Wiklund, 2006; Sales and Kotrba, 2013). In addition, some consumers prefer both natural and organic methods of production without the use of chemical soil fertilizers and only minimal processing. Several affluent consumer groups are also concerned about the animal production environment and are, therefore, interested in organic products as well as products obtained by natural (low-input system) methods (de Boer et al., 2014; Latvala et al., 2012). Wild animals enjoy well-being and unrestricted access to natural pastures, making their free choice of food.

Wild animals have been used as a protein source throughout man's evolutionary history. Recent statistical updates in Europe indicate a growing interest of consumers in game (Daszkiewicz, 2007; Hoffman and Wiklund, 2006; Quaresma et al., 2011). Due to its dietary value and specific desirable aroma and taste, game, including wild boar meat, has gained popularity among consumers worldwide, despite its high price (Sales and Kotrba, 2013). Polish wild boar meat is an exclusive raw material, mainly for export (Daszkiewicz, 2007; Hoffman and Wiklund, 2006; Żochowska et al., 2005).

The number of wild boars hunted for human consumption in Poland is relatively high. Between 2007 and 2008 approximately 149 000 wild boars were hunted, and in the years 2016 and 2017, the number was over 310 000 (Central Statistical Office, 2009, 2017). The largest number of boars shot in the country are individuals with a body weight of 50 kg with a 10 % tolerance. They are most often animals aged 12–18 months. Among the boars shot, the majority are female specimens. The composition of wild boar meat is different from pork due to different living conditions (Kasprzyk, 2012). The most important advantages of wild boar meat are undoubtedly sensory features and nutritional value (Sales and Kotrba, 2013). Due to the wild animals' living environment, game is classified as an organic food.

In general meat quality from wild boars depends on the season, feed resources as well as the living conditions and sexual activity of the animals. For example, after the autumn mating season, the occurrence of fatty meat is rare (Kasprzyk, 2012). Moreover, the structure and texture of wild boar muscles depend on age (Żochowska et al., 2005) and body weight. There is an apparent information gap in the literature regarding the physicochemical characteristics of wild boar meat of different body weight (Quaresma et al., 2011).

The purpose of the work was to assess the technological and nutritional quality of meat from female wild boars of south-eastern Poland.

## 2 Material and methods

### 2.1 Animals

The experiment was performed on meat of female wild boars. The animals were shot in south-eastern Poland during planned hunting in accordance with the regulations (Polish Parliament, 1995) concerning hunting by the hunting authorities. Wild animals were not killed specifically for this research, and, as such, no specific authorization was needed for tissue sampling according to the applicable national laws. The study was carried out by using tissues of free-living wild boar killed during regular hunting under applicable national laws. In all of the cases, sampling of tissues was performed during the slaughtering and dressing procedures. Therefore, our research does not require the approval of the Animal Experimentation Committee. Wild boars used in this research lived in the forest ecosystems. After being shot, the animals were gutted and then transported to a collection point, where they were weighed on calibrated scales, after which they were skinned and chilled.

The weight of carcasses of boars shot was in the range of 30–95 kg. Carcasses were allocated to five groups according to weights: group I – 30±5 kg; group II – 45±4.9 kg; group III – 60±4.7 kg; group IV – 75±5.2 kg; group V – 90±5.0 kg. Two muscles from 40 carcasses, i.e., 8 carcasses in each group, were taken for the experiment. The Musculus longissimus thoracis et lumborum (LTL) from the thoracic–lumbar section and Musculus semimembranosus (SM) were cut from refrigerated carcasses in the local meat processing plant Elite Expeditions Game Processing Base in Zwierzyniec. After removing the external fat, about 0.8 kg samples of meat were packed in plastic bags, kept refrigerated (approx. 4±2 ${}^{\circ }$C) and transported to the laboratory of the University of Life Sciences in Lublin.

### 2.2 Physicochemical properties

The ultimate meat pH was determined 48 h post mortem using the aqueous meat extract pH according to PN-ISO 2917:2001 (2001). Water holding capacity (WHC) of the meat was assayed by the sedimentation method (Wierbicki et al., 1962). Briefly, about 50 g of the minced meat was mixed with five parts of water at 4 ${}^{\circ }$C, mechanically homogenized for 1 min at the speed of 10${}^{4}$ min${}^{-1}$ (T25 Basic ULTRA-TURRAX, IKA, Staufen, Germany) and separated at 1500g for 20 min using a MPW-350R centrifuge (MPW Med-Instruments, Warszawa, Poland). WHC was determined on the basis of the amount of sediment. Drip loss was determined on the basis of weight changes of the meat portions after 4 d of storage at 4 ${}^{\circ }$C according to the method of Prange et al. (1977). Cooking loss was determined after heating the vacuum packed meat samples in a water bath to 75 ${}^{\circ }$C in the center (Honikel, 1998). The CIE $L^{*}a^{*}b^{*}$ color parameters of the meat were determined using an X-Rite Series 8200 spherical spectrophotometer with a diameter of 12.7 mm and D 65/10${}^{\circ }$ standard illuminator. Color parameters were measured on a freshly cut surface of each sample wrapped in transparent 23 µm foil following the recommendations of the American Meat Science Association (2012). The myoglobin content of the meat was analyzed according to the Krzywicki (1982) method with the Tang et al. (2004) modification. The test was based on the absorbance of the extract in a cold 40 mM phosphate extraction buffer. The meat frozen at -24±2 ${}^{\circ }$C for 1 week was ground, homogenized in a phosphate buffer of pH 6.8 and separated by centrifugation at 1500g. By means of reflectance values read at 572, 565, 545 and 525 nm in a UV–visible spectrophotometer (Nicolet Evolution 300 BB, Thermo Fisher Scientific, Waltham, USA), the total myoglobin content and the proportions of myoglobin, oxymyoglobin and metmyoglobin were calculated from the Tang et al.'s (2004) formula. The assessment of the mechanical properties of cooked samples (after cooking loss determination) cooled to room temperature was carried out with the TA.XT. plus texture analyzer (Stable Micro Systems) equipped with the 50 N head, HDP/90, HDP/WBV and P/100 attachments. The speed of deformation was 1 mm s${}^{-1}$. A rectangle (15×15×75 mm) was used for the shear test and cylindrical (ø 15×10 mm) samples for compression. Muscle cutting was done across muscle fiber, and compression was done parallel to the muscle fibers (Bourne, 2002). Data were collected with Texture Expert Exceed software (Stable Micro Systems). The basic chemical composition was assayed according to AOAC standards (Association of Official Analytical Chemists, 2000). The caloricity of meat was calculated using Atwater values (Mansour and Khalil, 1999). Physicochemical analyses were performed in quadruplicate in each experiment.

### 2.3 Statistical analyses

The obtained results were grouped and submitted to statistical calculations, presenting the arithmetic averages of each of the examined features and the values of the standard deviation (SD). In the calculation, the method of two-way analysis of variance (ANOVA) was used (carcass weight and type of muscle as well as their interactions were considered as fixed effects), and the significance of differences between the means was determined using the Tukey's test at the significance level P<0.05 and P<0.01. The calculations were carried out using the Statistica software, version 13.0 (StatSoft, Kraków, Poland).

## 3 Results and discussion

The physicochemical characteristics of raw meat affect its appearance and sensory quality (Daszkiewicz, 2007). According to The National Producers Council (NPPC) the most important parameters for the consumers of pork meat are natural drip loss, color, pH, intramuscular fat (IMF) content, tenderness and taste (NPPC Pork Quality Solutions Team, 2006).

The decline in pH following death caused by lactic acid accumulation is one of the most remarkable factors of muscle transformation in meat, with a crucial importance in its future quality (Robergs et al., 2004). The pH value is a factor which has a direct influence on the technological properties of meat. According to Binder et al. (2004) eating quality of the loin is most desirable at intermediate pH (5.4–6.0). In this study the pH value (Table 1) ranged from 5.62 to 5.66 for LTL and from 5.62 to 5.68 for SM proving normal glycolysis and glycogenolysis progress in all analyzed groups (Wiklund and Smulders, 2011). The value of pH${}_{48}$ of female wild boar meat was typical for game (Stanisz et al., 2019). pH values similar to the present data were recorded for wild boars by Cifuni et al. (2014). In turn, Müller et al. (2000) detected a pH${}_{24}$ of 5.45 in wild boars hunted in Germany.

**Table 1:** Technological value of meat from female wild boars (*Sus scrofa scrofa* L. NS: nonsignificant.) of different carcass weights.

Parameter		Group					Effect		
	Muscle	I	II	III	IV	V	Muscle	Group	Muscle × group
		Mean ± SD	Mean ± SD	Mean ± SD	Mean ± SD	Mean ± SD			
pH	LTL	$5.63^{\mathrm{ab}}\pm 0.05$	$5.66^{\mathrm{aA}}\pm 0.03$	$5.63^{b}\pm 0.01$	$5.64^{a}\pm 0.01$	$5.62^{B}\pm 0.02$			
	SM	$5.68^{\mathrm{ab}}\pm 0.14$	$5.62^{a}\pm 0.02$	$5.66^{b}\pm 0.01$	$5.67^{b}\pm 0.01$	$5.62^{a}\pm 0.02$	NS	${}^{**}$	NS
WHC (%)	LTL	20.19±0.78	20.10±1.07	24.48±0.64	20.80±1.21	20.94±1.54			
	SM	$20.74^{C}\pm 1.38$	$21.89^{b}\pm 0.85$	$22.35^{B}\pm 0.22$	$22.79^{\mathrm{aAB}}\pm 0.78$	$23.09^{A}\pm 0.35$	${}^{**}$	${}^{**}$	NS
Drip loss (%)	LTL	$3.03^{\mathrm{ab}}\pm 2.02$	$3.19^{a}\pm 1.46$	$2.97^{a}\pm 0.64$	$1.79^{b}\pm 0.52$	$2.55^{a}\pm 0.71$			
	SM	$3.54^{a}\pm 1.86$	$3.45^{a}\pm 0.54$	$1.77^{b}\pm 0.35$	$3.06^{a}\pm 0.20$	$3.19^{a}\pm 0.24$	NS	NS	NS
Cooking loss (%)	LTL	$35.27^{a}\pm 3.39$	$35.71^{a}\pm 0.22$	$34.01^{a}\pm 0.82$	$33.98^{a}\pm 0.28$	$32.01^{b}\pm 0.55$			
	SM	36.71±4.10	34.90±1.20	34.72±2.21	33.53±2.00	33.92±1.11	NS	NS	NS
CIE $L^{*}$	LTL	$45.41^{A}\pm 1.80$	$45.20^{A}\pm 0.80$	$41.51^{B}\pm 1.30$	$41.31^{B}\pm 1.00$	$41.13^{B}\pm 1.40$			
	SM	$40.74^{a}\pm 2.1$	39.60±1.00	39.31±0.90	39.22±1.10	$38.33^{b}\pm 2.10$	${}^{**}$	${}^{**}$	NS
CIE $a^{*}$	LTL	$9.10^{b}\pm 1.00$	$10.30^{a}\pm 1.10$	$12.41^{a}\pm 3.01$	$11.51^{a}\pm 0.40$	$11.10^{a}\pm 1.10$			
	SM	$10.60^{B}\pm 1.10$	11.30±1.30	11.27±0.70	11.35±0.70	$12.62^{A}\pm 1.00$	NS	${}^{**}$	NS
CIE $b^{*}$	LTL	$10.60^{a}\pm 1.00$	$10.3^{a}\pm 1.00$	$10.31^{a}\pm 1.60$	$10.2^{a}\pm 0.50$	$8.81^{b}\pm 1.00$			
	SM	$10.20^{\mathrm{bC}}\pm 1.10$	$9.60^{B}\pm 0.40$	$9.30^{\mathrm{aB}}\pm 0.80$	$9.04^{B}\pm 0.90$	$8.31^{A}\pm 0.90$	${}^{**}$	${}^{**}$	NS

Means in the same row with different letters are significantly different: ${}^{a,\;b}$ P≤0.05; ${}^{A,\;B,C}$ P≤0.01. LTL: M. longissimus thoracis et lumborum; SM: M. semimembranosus; WHC: water holding capacity. Group I – 30±5 kg; group II – 45±4.9 kg; group III – 60±4.7 kg; group IV – 75±5.2 kg; group V – 90±5.0 kg.

The ability to bind water is one of the most important factors shaping the quality and economic value of meat. This feature affects the juiciness and tenderness of meat, as well as changes in water content in meat during transport, storage and thermal processing (Irie et al., 1996). In our study there were statistically significant (P≤0.01) differences in the WHC of the SM muscles between the lowest-weight carcasses compared to the heavier carcasses. A higher WHC value of heavy-weight carcasses offers better technological efficiency. Convergent results regarding WHC were noted in wild boars by Żmijewski and Korzeniowski (2001). Some studies suggest that there is a relationship between post mortem proteolysis and water holding in meat (Huff-Lonergan and Lonergan, 2005; Zeng et al., 2017). However, the exact mechanisms determining the WHC of meat are not fully understood.

Drip loss changes the chemical composition and affects the acceptance (quality) of meat (Otto et al., 2007). The recorded values of drip loss in LTL and in SM ranged from 1.79 % to 3.03 % and from 1.77 % to 3.54 %, respectively, and were characteristic of normal meat (Marchiori and Felício, 2003). Drip loss decreased with increasing mass of carcass, and this trend was similar to that observed in the case of the Brazilian wild boar meat as reported by Marchiori and de Felício (2003) and was close to data reported by Wiklund and Smulders (2011). Similar findings were observed by Batorska et al. (2018) in wild boar meat.

The next analyzed feature was cooking loss. In the LTL muscle, statistically significant differences between group V and the other groups were noted. With respect to this feature, no statistically significant differences were noted in the SM muscle. Similar values for this trait were recorded by Stanisz et al. (2019) when analyzing the semimembranosus muscle of this species.

The color of meat is an extremely important factor that influences a consumer's purchase decision as it is deemed a visual measure of freshness and quality (Khliji et al., 2010). Carrasco et al. (2009) have reported that meat color may be influenced by several factors, such as enzymes, diet and age of the animal and even the activity undertaken by the animal. Analyzing the value of the $L^{*}$ parameter, it was found that it was typical for game (Sales and Kotrba, 2013; Cifuni et al., 2014; Pedrazzoli et al., 2017). There were statistically significant differences (P≤0.01) in $L^{*}$ parameter values in the LTL muscle between groups I and II and the other groups. The maximum difference in the lightness the LTL in groups I and V is above 4 points. Regarding SM, significant differences (P≤0.05) in muscle lightness occurred between groups I and V. The values of parameter CIE $a^{*}$ were similar to those presented by Sales and Kotrba (2013) but higher than those obtained by Hoffman and Sales (2007). The color of meat primarily depends on the concentration and chemical forms of myoglobin, which is responsible for oxygen transport in muscles (Ruusunen and Puolanne, 2004; Skewes et al., 2014). The concentration of hem-pigment usually increases with the age and weight of animals (Kołacz, 2007), and that trend was confirmed by our study.

There were statistically significant differences in the content of myoglobin in LTL and SM between the analyzed groups (Table 2). The weight of the carcasses of wild boar significantly affected the content of dyes in the extract and tissue. It can be assumed that a higher pigment content observed in the highest carcasses is associated with the higher activity and increased oxidative metabolism in adults and older animals. This explains the increased redness of the meat (Ruusunen and Puolanne, 2004; Sales and Kotrba, 2013; Skewes et al., 2014). The content of myoglobin in muscles composed of more red muscle fibers and the oxidative/glycolytic type, according to Lindahl (2005), is higher than in muscles with a predominance of white muscle fibers. There are more capillaries around the type I and IIA fibers than around the type IIB fibers (Ruusunen and Puolanne, 2004). As a result of systematic “training”, muscle fibers undergo transformation processes, and there is a reduction in type IIB (white) fiber in favor of oxidation glycolytic and oxidative (Bogucka et al., 2008; Daszkiewicz, 2007). Mature wild boars presented higher percentages of fibers I and IIB compared to young animals (Skewes et al., 2014). The muscles of animals living in the wild and focusing intensively on seeking food work continuously, thus mainly exciting the oxidative metabolism (Bogucka et al., 2008). It can also be presumed that the greater amount of water observed in the lower-weight carcasses affected the dilution of dyes. During the course of storage in air, myoglobin is oxygenated to form red oxymyoglobin, which is gradually oxidized by oxygen in the air to form unattractive brown–red metmyoglobin (Kołacz, 2007). The change in color of fresh meat depends on the rate of pigment oxidation and the efficiency of the enzymatic reduction process of metmyoglobin and is the first indicator of deterioration (Daszkiewicz, 2007). A higher storage temperature and lower pH speed up oxidation, while metal ions and light also speed up the reactions (Hofbauer and Smulders, 2011).

**Table 2:** Pigment content in meat from female wild boars (*Sus scrofa scrofa* L.) of different carcass weights.

Parameter		Group					Effect		
	Muscle	I	II	III	IV	V	Muscle	Group	Muscle × group
		Mean ± SD	Mean ± SD	Mean ± SD	Mean ± SD	Mean ± SD			
Myoglobin (%)	LTL	$20.00^{C}\pm 0.92$	$20.02^{C}\pm 0.70$	$21.81^{B}\pm 0.60$	$21.83^{B}\pm 0.50$	$24.41^{A}\pm 0.31$			
	SM	$16.61^{D}\pm 0.93$	$20.00^{C}\pm 0.50$	$20.04^{C}\pm 0.20$	$22.32^{B}\pm 0.30$	$25.14^{A}\pm 0.40$	${}^{**}$	${}^{**}$	NS
Oxymyoglobin (%)	LTL	$49.61^{A}\pm 5.98$	$40.39^{B}\pm 6.78$	$47.66^{A}\pm 3.42$	$46.75^{A}\pm 2.81$	$40.33^{B}\pm 1.03$			
	SM	$36.62^{A}\pm 2.07$	38.77±1.10	35.68±1.62	34.46±2.04	$31.47^{B}\pm 1.33$	${}^{**}$	${}^{**}$	NS
Metmyoglobin (%)	LTL	$30.40^{B}\pm 5.13$	$39.61^{A}\pm 6.45$	$30.58^{B}\pm 3.21$	$31.45^{\mathrm{bB}}\pm 4.56$	$35.30^{\mathrm{aA}}\pm 2.13$			
	SM	$46.77^{A}\pm 0.89$	$41.23^{C}\pm 1.60$	$44.32^{B}\pm 1.35$	$43.22^{B}\pm 0.99$	$43.39^{B}\pm 0.82$	${}^{**}$	${}^{**}$	NS
Pigment content in	LTL	$21.82^{C}\pm 9.10$	$25.90^{C}\pm 1.10$	$28.03^{C}\pm 3.90$	$47.92^{B}\pm 3.01$	$56.80^{A}\pm 4.00$			
extract (µmol L${}^{-1}$)	SM	$18.52^{D}\pm 0.4$	$24.91^{C}\pm 0.40$	$27.20^{B}\pm 1.92$	$27.63^{B}\pm 0.73$	$29.70^{A}\pm 0.54$	${}^{**}$	${}^{**}$	NS
Pigment content in	LTL	$4.29^{\mathrm{ab}}\pm 2.29$	$5.01^{b}\pm 0.33$	$5.95^{\mathrm{aC}}\pm 0.99$	$9.49^{B}\pm 0.42$	$13.68^{A}\pm 0.37$			
tissue (mg g${}^{-1}$)	SM	$3.44^{C}\pm 0.03$	$4.72^{B}\pm 0.08$	$5.35^{B}\pm 0.99$	$5.57^{B}\pm 0.04$	$5.70^{A}\pm 0.10$	${}^{**}$	${}^{**}$	NS

Means in the same row with different letters are significantly different: ${}^{a,\;b}$ P≤0.05; ${}^{A,\;B}$ P≤0.01. LTL: M. longissimus thoracis et lumborum; SM: M. semimembranosus. Group I – 30±5 kg; group II – 45±4.9 kg; group III – 60±4.7 kg; group IV – 75±5.2 kg; group V – 90±5.0 kg.

The next analyzed parameters were shear force and texture profile analysis (TPA) presented in Table 3. These features are particularly important when assessing the value of meat intended for culinary purposes. The type of muscle significantly affected the shear force. Meat from the LTL muscle was more tender. There were significant differences (P≤0.01) in the shear force of the LTL muscle between groups I and II and IV and V. Meat from group V in comparison with group I showed a higher shear force of 18 N (LTL) and 15 N (SM). In the SM muscle no statistically significant differences in shear force were observed. In relation to hardness, gumminess and resilience, significant differences were noted (P≤0.01) between groups IV and III, V. Between the above groups significant differences (P≤0.05) occurred also in chewiness. A higher tenderness of meat from the carcass of wild boars was also observed by Żmijewski and Korzeniowski (2001) as well as Żochowska et al. (2005). The increase in the shear force values of meat from a higher carcass weight observed in our research, as compared to lower-weight carcasses, may result from the reduction in muscle proteolysis (Kim et al., 2009) as well as the differences in collagen content (Hofbauer and Smulders, 2011). According to Żochowska et al. (2005) less tenderness may result from a higher fiber cross-sectional area and the thickest connective tissue of perimysium and endomysium.

**Table 3:** Texture parameters of meat from female wild boars (*Sus scrofa scrofa* L.) of different carcass weights.

Parameter		Group					Effect		
	Muscle	I	II	III	IV	V	Muscle	Group	Muscle × group
		Mean ± SD	Mean ± SD	Mean ± SD	Mean ± SD	Mean ± SD			
Shear force (N)	LTL	$52.20^{B}\pm 6.10$	$52.71^{B}\pm 7.20$	$60.22^{a}\pm 10.90$	$67.03^{\mathrm{aA}}\pm 11.11$	$70.20^{\mathrm{aA}}\pm 10.30$			
	SM	69.50±4.15	74.01±5.61	78.42±16.1	81.93±15.9	84.40±11.70	${}^{**}$	${}^{**}$	NS
Hardness 1	LTL	143.80±17.30	145.45±11.64	$119.53^{B}\pm 12.10$	$171.90^{A}\pm 0.01$	$121.08^{B}\pm 10.38$			
	SM	$108.60^{b}\pm 24.53$	141.41±16.62	139.63±22.56	$164.75^{a}\pm 24.72$	144.34±14.94	NS	${}^{**}$	NS
Hardness 2	LTL	131.95±16.10	132.24±10.58	$108.77^{B}\pm 11.65$	$158.62^{A}\pm 1.21$	$111.40^{B}\pm 14.01$			
	SM	$99.71^{b}\pm 22.89$	132.45±0.85	129.42±23.10	$153.90^{a}\pm 23.22$	134.24±15.36	NS	${}^{**}$	NS
Cohesiveness	LTL	0.57±0.02	0.57±0.01	0.56±0.02	0.57±0.02	0.58±0.01			
	SM	0.58±0.02	$0.62^{a}\pm 0.02$	$0.57^{b}\pm 0.03$	0.61±0.01	0.61±0.01	${}^{**}$	${}^{*}$	NS
Springiness	LTL	0.53±0.02	0.54±0.01	0.52±0.03	0.52±0.04	0.53±0.04			
	SM	0.52±0.05	0.56±0.03	0.55±0.04	0.55±0.02	0.52±0.06	NS	NS	NS
Gumminess	LTL	81.94±9.33	83.99±8.78	$67.79^{B}\pm 8.79$	$99.86^{A}\pm 11.42$	$70.26^{B}\pm 8.51$			
	SM	$63.68^{b}\pm 15.92$	87.81±11.38	80.53±17.47	$99.62^{a}\pm 15.83$	87.68±9.22	NS	${}^{**}$	NS
Chewiness	LTL	43.75±3.99	45.21±5.07	$35.43^{b}\pm 3.48$	$52.37^{a}\pm 10.60$	$37.52^{b}\pm 3.16$			
	SM	33.44±9.90	48.85±6.02	44.61±13.63	55.03±10.22	45.99±9.52	NS	${}^{*}$	NS
Resilience	LTL	$0.19^{B}\pm 0.01$	0.21±0.01	$0.20^{B}\pm 0.01$	$0.23^{A}\pm 0.01$	$0.19^{B}\pm 0.01$			
	SM	0.19±0.03	0.321±0.02	0.20±0.02	0.23±0.02	0.23±0.02	NS	${}^{**}$	NS
Stringiness	LTL	10.66±0.64	10.37±0.42	11.22±0.72	10.36±0.22	10.92±0.86			
	SM	10.04±0.92	10.07±0.40	9.95±0.66	10.24±0.90	9.81±0.58	${}^{*}$	NS	NS

Means in the same row with different letters are significantly different: ${}^{a,\;b}$ P≤0.05; ${}^{A,\;B}$ P≤0.01. LTL: M. longissimus thoracis et lumborum; SM: M. semimembranosus. Group I – 30±5 kg; group II – 45±4.9 kg; group III – 60±4.7 kg; group IV – 75±5.2 kg; group V – 90±5.0 kg.

Table 4 shows numerical data presenting the content of the main chemical components in the LTL and SM. The carcasses included in group I were characterized by a statistically (P≤0.01) lower protein content compared to the other groups, similar to that described by Sales and Kotrba (2013). The protein content in wild boar meat in our study was similar to those recorded in the sirloin and ham muscles of wild boars by Żmijewski and Korzeniowski (2001). A higher protein content in wild boar meat was noted by Batorska et al. (2018) and Stanisz et al. (2019). The meat contains a protein of high biological value. It is conditioned by the presence of all essential exogenous amino acids necessary both in terms of nutrition as well as by construction of structures and a proper balancing of the body (Vaclavik and Christian, 2008). The meat protein is digestible to about 92 %–94 % (Ahmad et al., 2018; Williams, 2007).

**Table 4:** Nutritional value of meat from female wild boars (*Sus scrofa scrofa* L.) of different carcass weights.

Parameter		Group					Effect		
	Muscle	I	II	III	IV	V	Muscle	Group	Muscle × group
		Mean ± SD	Mean ± SD	Mean ± SD	Mean ± SD	Mean ± SD			
Total protein	LTL	$21.42^{B}\pm 0.20$	$21.80^{A}\pm 0.20$	$21.81^{A}\pm 0.11$	$21.82^{A}\pm 0.10$	$21.84^{A}\pm 0.20$			
content (%)	SM	$21.11^{B}\pm 0.21$	$21.40^{A}\pm 0.20$	$21.53^{A}\pm 0.22$	$21.53^{A}\pm 0.21$	$21.60^{A}\pm 0.20$	${}^{**}$	${}^{**}$	NS
Intramuscular fat	LTL	$1.46^{C}\pm 0.13$	$1.91^{B}\pm 0.13$	$2.01^{\mathrm{bB}}\pm 0.15$	$2.12^{A}\pm 0.14$	$2.20^{\mathrm{aA}}\pm 0.16$			
(%)	SM	$1.80^{\mathrm{cC}}\pm 0.23$	$2.13^{\mathrm{cB}}\pm 0.33$	$2.46^{b}\pm 0.27$	$2.69^{A}\pm 0.28$	$2.79^{\mathrm{aA}}\pm 0.26$	${}^{**}$	${}^{**}$	NS
Energy value	LTL	$136.00^{B}\pm 4.41$	$142.60^{A}\pm 5.40$	$143.3^{A}\pm 6.70$	$144.40^{A}\pm 7.60$	$144.43^{A}\pm 5.61$			
(kcal 100 g${}^{-1}$)	SM	$137.40^{B}\pm 5.40$	$142.70^{A}\pm 5.90$	$145.23^{A}\pm 6.80$	$147.50^{A}\pm 7.21$	$147.70^{A}\pm 5.40$	NS	NS	NS
Water content (%)	LTL	$74.60^{A}\pm 0.20$	$74.31^{B}\pm 0.21$	$74.22^{B}\pm 0.20$	$74.30^{B}\pm 0.11$	$74.21^{B}\pm 0.10$			
		$74.20^{a}\pm 0.20$	$73.94^{b}\pm 0.30$	$73.93^{b}\pm 0.21$	$73.90^{b}\pm 0.22$	$73.91^{b}\pm 0.30$	NS	${}^{**}$	${}^{*}$

Means in the same row with different letters are significantly different: ${}^{a,\;b}$ P≤0.05; ${}^{A,\;B}$ P≤0.01. LTL: M. longissimus thoracis et lumborum; SM: M. semimembranosus. Group I – 30±5 kg; group II – 45±4.9 kg; group III – 60±4.7 kg; group IV – 75±5.2 kg; group V – 90±5.0 kg.

Fat is an elementary factor in the dietary value of meat. At the point of purchase, the amount of visible fat is the strongest visual discriminative stimulus in the decision making process. Fatty meat was regarded by consumers as unhealthy, which is why the market demand is for lean meat without fat cover (Jukna and Valaitiene, 2012). However, the optimal amount of intramuscular fat and the loosening of the connective tissue are necessary in order to favorably shape the sensory characteristics of meat, i.e., the tenderness, tastiness and juiciness of meat (Frank et al., 2016). In addition, the fat reduces the amount of losses during the heat treatment because an adequate fact content has a positive effect on the ability to keep water in meat. In this study the lowest IMF content was found in the low-weight carcasses. The observed differences in IMF content between group I and other analyzed groups were statistically significant. Fat increases with increasing body weight (Kasprzyk, 2012). This is due to the fact that the muscles and bones develop first as the animal grows, and later the formation of adipose tissue occurs. The range of variability of this feature in our own research was similar to the results recorded for wild boar by Żmijewski and Korzeniowski (2001) and Batorska et al. (2018) and was located in the range of optimal values in relation to the fat content. Most researchers dealing with the issue of meat quality state that for good-quality meat, the IMF content should be within 2 %–3 %. The fat content in the meat of wild boars in our study was lower than its content in pork reported by Tikk et al. (2007; 2.5 %–2.9 %) and by Choi et al. (2012; 4.97 %).

A higher fat and protein content causes a significantly higher energetic value of the meat. The caloricity for group V of LTL and SM muscles was higher by 8.4 and 10.3 kcal, respectively, in comparison with group I. Regarding the energy value of meat, Żmijewski and Korzeniowski (2001) concur with the results noted in our research. Therefore, the meat of wild boar is an interesting and attractive component of the diet due to its low calorie content.

Statistically significant differences (P≤0.01) were noted in relation to the water content in LTL between group I and the other groups. Significantly lower water content in SM was noted for carcasses from groups II, III, IV and V in comparison with carcasses from group I. A much higher water content was characteristic for boar meat assessed by Stanisz et al. (2019), while Batorska et al. (2018) noted a lower content.

## 4 Conclusions

Summing up, both the carcass weight and the muscle of wild boars affected the physicochemical properties of meat, causing differences in the WHC, shear force, meat brightness, content of hem-dyes, protein, fat and water, thus changing the technological usability and nutritional properties of meat. The group's effect on muscle pH was noted, while the type of muscle had no effect on this trait. Generally, wild boar meat can be used as a culinary meat, while lighter carcasses have better nutritional value.

## Data Availability

The data sets are available upon request from the corresponding author.
